# Sleep Disturbances in Panic Disorder with Comorbid Complex PTSD: A Possible Relationship and Different Psychopathology?

**DOI:** 10.3390/life13081636

**Published:** 2023-07-27

**Authors:** Elvira Anna Carbone, Giulia Menculini, Renato de Filippis, Martina D’Angelo, Leonardo Zebi, Luca Steardo

**Affiliations:** 1 Department of Medical and Surgical Sciences, University “Magna Græcia”, 88100 Catanzaro, Italy; elvira.carbone@unicz.it; 2 Department of Psychiatry, University of Perugia, Piazzale Lucio Severi, 1, 06132 Perugia, Italy; giulia.menculini@unipg.it (G.M.); leonardo.zebi@libero.it (L.Z.); 3 Department of Health Sciences, University “Magna Græcia”, 88100 Catanzaro, Italy; defilippisrenato@gmail.com (R.d.F.); martina.dangelo001@studenti.unicz.it (M.D.)

**Keywords:** complex post-traumatic stress disorder, panic disorder, sleep disturbances, anxiety, hypnotic drug use

## Abstract

Background: Several studies have shown the possible link between trauma and sleep disturbances, particularly in anxiety disorders. This issue could be because sympathetic hyperarousal is central to both disorders, probably caused by a dysregulation of the noradrenergic system. This study aimed to establish if the comorbidity with complex post-traumatic stress disorder (cPTSD) is associated with sleep disturbances in panic disorder (PD) and if the presence of poor sleep quality is associated with a higher psychopathological burden. Methods: Participants (N = 211) with PD completed the International Trauma Questionnaire concerning their most troubling experience, the Hamilton Anxiety Rating Scale (HAM-A), and the Pittsburgh Sleep Quality Index (PSQI) to assess anxiety symptoms and sleep disturbances, respectively. Results: The sample was divided into two subgroups based on the presence of cPTSD. No significant differences emerged in the bivariate analyses for what concerns sociodemographic features. As for the scores of the psychopathological scales, the analysis highlighted statistically significant differences between the subgroups. Subjects with cPTSD reported significantly higher HAM-A total scores. As for the disturbances in self-organization (DSO) and PSQI scores, these were all significantly higher in the cPTSD subsample. At the logistic regression, the presence of cPTSD was inserted as the dependent variable, while the PSQI scores of the subscales evaluating subjective sleep quality, sleep duration, sleep efficacy, and the use of hypnotics were used as independent variables. The presence of cPTSD was significantly associated with the PSQI subscores for subjective sleep quality and use of hypnotics. Conclusions: Patients with PD exhibit more severe sleep disturbances and a higher anxiety burden when experiencing prolonged trauma. Therapeutic advances are needed in this field to target these symptomatologic domains.

## 1. Introduction

Traumatic experiences are very common in people who develop a psychiatric disorder, and exposure to childhood trauma has been recognized as a predictor of the worst outcomes in most of these conditions [1,2]. Subsequently, the effects of trauma and chronic exposure to traumatic events could deserve interest not only concerning specific accidents or childhood maltreatment but also in daily life [3]. In this regard, the ICD-11 outlined two “trauma-related disorders”, namely post-traumatic stress disorder and complex post-traumatic stress disorder (PTSD and cPTSD), with a different categorization than that proposed in the prior versions of both the ICD and the Diagnostic and Statistical Manual—5th Edition (DSM-5) [4]. PTSD and cPTSD are characterized by re-experiencing and avoiding traumatic events and feeling constantly threatened. The lifetime prevalence of PTSD ranges from 3 to 9% in adult population based on type and number of traumas; cPTSD prevalence ranges from 1 to 8% in general population, increasing up to 50% in mental health facilities [5,6,7]. The main difference between cPTSD and PTSD is the protracted psychological changes that trauma can bring back and lead to chronic symptomatology. [8]. Moreover, particular attention has been paid to the disturbance in self-organization that often occurs in this population. The symptomatology termed “disturbances in self-organization” (DSO) has been divided into three clusters of clinical features: affective dysregulation, negative self-concept, and disturbed relationships [9]. Based on the taxonomic structure of ICD-11, PTSD and cPTSD cannot be diagnosed at the same time. cPTSD is more frequently associated with reiterated and protracted trauma, but the type of exposure is a risk factor rather than a differential diagnostic requisite [10]. Trauma exposure can happen both during childhood and adulthood, but interpersonal trauma, which is trauma perpetrated by a person or people to harm others, denotes a particularly significant risk factor for cPTSD when occurring during early development [11].

Among anxiety disorders affecting up to 301 million people worldwide (4.05% global prevalence) [12] and representing the most common psychiatric condition worldwide with high comorbidity rates [13], panic disorder (PD) has been described as the most severe form. It affects around 2–5% of the population [14,15,16,17], usually occurring without a specific trigger, but seriously impacting the overall functioning of the affected individuals [18]. The pathogenesis of PD has received increasing attention, and traumatic experiences, particularly those occurring during childhood, were pointed out as one of the main risk factors for this condition [19]. Traumatic experiences and the development of panic have been historically linked. Indeed, it was reported that up to 69% of subjects seeking treatment for PTSD who experienced trauma also met the criteria for PD [20]. Conversely, patients with PD often report histories of traumatic experiences [21]. To note, sympathetic hyperarousal is central to both disorders, and in both, dysregulation of α_2_ auto-receptors in the locus coeruleus has been hypothesized. As noted previously, there are striking similarities between the flashbacks of PTSD and the panic attacks of PD [22].

Despite these findings, PTSD is often undetected and untreated in PD. Indeed, many patients were frequently identified as suffering from anxiety or panic disorder rather than PTSD [23]. This could at least partially depend on the wide range of PTSD symptoms [24] that may be a challenge for differential diagnosis as well as on a propensity for clinicians to not assess traumatic stress symptoms fully and routinely during clinical evaluation. The detection of features related to the post-traumatic spectrum would be of utmost importance in this population due to the possible higher clinical severity underpinned by this association [25].

Both PTSD and PD exhibit sleep disturbances [26]. Evidence prove that insomnia is more frequent in patients suffering from PD than in control populations [27,28] and that impairment in initiating and maintaining sleep may be influenced by sleep panic, probably due to dysregulation of arousal [29]. Moreover, the link between trauma and PD could underpin alterations in central fear systems, which could be consistent with their phenomenology and circadian rhythm abnormalities [30]. On the other hand, sleep disturbances are one of the DSM criteria and noticeable symptoms of PTSD [31]. Insomnia is frequently reported during the early aftermath of trauma, and it may be predictive of later developing PTSD [32,33]. Among symptom domains, it has been also demonstrated that sleep disturbances are particularly prevalent in subjects suffering from cPTSD when compared to those with PTSD, with insomnia showing up to 18-times higher frequency [34]. Former research underlined that PTSD and PD are both characterized by subjective difficulties in the initiation and maintenance of sleep and, in a substantial percentage of cases, by episodic parasomnias, namely trauma-related nightmares, and nocturnal panic attacks [35,36]. As consequence, alteration in sleep quality and duration may have a negative impact on quality of life, reducing performance or daytime wellbeing [37], and represents a risk factor for the early development of both disorders, maintenance, and treatment difficulties [38,39,40].

Scant evidence is available concerning the relationship between cPTSD and PD, but recent research demonstrated that subjects who childhood adverse childhood events and suffered from cPTSD also showed a higher prevalence of PD, even when compared with those affected by PTSD [41]. Thus, in the context of this comorbidity, sleep disorders could significantly worsen, leading to a higher risk of becoming chronic and experiencing treatment resistance [42].

This study aimed to establish if the comorbidity with cPTSD is associated with sleep disturbances in PD and if the presence of poor sleep quality is associated with a higher psychopathological burden. We hypothesized that participants with PD and cPTSD would report a greater symptom burden compared with those without cPTSD, with a higher prevalence of sleep impairments that would seriously affect psychological well-being. Since sleep disturbances represent a very impactful symptom domain in both conditions, we believe that unraveling this association would help optimize cPTSD treatment strategies in the context of this comorbidity.

## 2. Materials and Methods

### 2.1. Participants

This cross-sectional study was conducted in a naturalistic setting between 1 April 2020 and 31 July 2022. Participants were consecutively enrolled at the outpatient service of the Psychiatric Unit of the University Magna Graecia of Catanzaro. Patients were included in the study if they met the following criteria: (1) age between 18 and 75 years; (2) diagnosis of PD according to the Diagnostic and Statistical Manual of Mental Disorders-fifth edition (DSM-5); (3) willingness to participate in the study; and (4) current treatment with selective serotonin reuptake inhibitors (SSRI) or SSRI and benzodiazepines (BDZ) as recommended. We decided to include only subjects under appropriate pharmacological treatment for their condition in order to not bias the overall symptom severity. Exclusion criteria were: (1) inability to provide written informed consent; (2) moderate or severe cognitive impairment evaluated with Mini-Mental State Evaluation (MMSE) (cut-off > 25, (3) any neurologic disease or substance and/or alcohol-use disorder in comorbidity; and (4) being pregnant or in the postpartum period. All patients gave their written informed consent to participate in the study after a complete description of the study’s aims and design. The study was carried out following the latest version of the Declaration of Helsinki and was approved by the Local Ethics Committee (identifier: 307/2020). A statistical power analysis was performed with Gpower 3.1 to estimate the sample size; given an alpha error of 0.5, power of 0.95, and an effect size of 0.5, the projected sample size required was a minimum of 176 participants.

### 2.2. Procedure

Socio-demographic and clinical characteristics were recorded with an ad hoc schedule, which was filled in by trained clinicians during one single study visit. The diagnoses of PD and possible previous psychiatric comorbidities were conducted according to the DSM-5 criteria using the Structured Interview for DSM-5 Disorders, Clinician Version (SCID-5-CV) [43]. All participants diagnosed with PD were invited to complete a battery of tests to assess the most troubling trauma experiences, anxiety symptomatology, and sleep quality and disturbances. The whole sample was divided into two subgroups based on the presence of the diagnosis of cPTSD.

### 2.3. Measures

Participants answered the following tools:

- International Trauma Questionnaire (ITQ) [44,45].

The ITQ focuses on the basic characteristics of PTSD and cPTSD and was developed following the diagnostic principles of ICD-11 [46]. The ITQ evaluates how much the patient has been disturbed by six core PTSD symptoms in the past month using a five-point Likert scale ranging from 0 (“*Not at all*”) to 4 (“*Extremely*”). Two symptoms consider the re-experiencing cluster of symptoms (upsetting dreams and flashbacks); two the avoidance cluster (avoiding internal or external reminders); and two the sense of current threat cluster (being hypervigilant or easily startled). A set of six questions evaluate disturbances in self-organization (DSO) symptoms to detect a probable cPTSD. Two items reflect each of the three clusters: (1) “affective dysregulation”; (2) “negative self-concept”; and (3) “disturbed relationships”. Additional items investigate functional impairment associated with both PTSD and DSO symptoms in three domains: (1) relationships and social life; (2) work or ability to work; and (3) other important aspects of life. Cronbach’s alpha (α) for the current study was 0.94.

Participants were considered as fulfilling the criteria for probable ICD-11 PTSD if qualifying trauma was reported and a score of ≥2 (“Moderately”) achieved for at least one of two symptoms from each of the respondents indicating the frequency of symptoms in the prior 2 weeks on a four-point Likert-scale, ranging from 0 (not at all) to 3 (nearly every day). Total scores range from 0 to 27. A probable cPTSD was diagnosed if patients fulfilled the criteria for PTSD in addition to scoring ≥2 (“Moderately”) for at least one symptom from each DSO cluster, including functional impairment associated with these symptoms [46].

- Hamilton Anxiety Rating Scale (HAM-A) [47].

This tool is extensively used in both clinical and research settings. The scale consists of 14 items, each defined by a series of symptoms, and measures both psychic anxiety (mental agitation and psychological distress) and somatic anxiety (physical complaints related to anxiety).

- Pittsburgh Sleep Quality Index (PSQI) [48].

This is a self-rated questionnaire that assesses sleep quality and disturbances over a 1-month time interval. The 19 items produce seven subscales: subjective sleep quality, sleep latency, sleep duration, habitual sleep efficiency, sleep disturbances, use of sleeping medication, and daytime dysfunction. The global score is the sum of these seven subscales scores.

### 2.4. Data Analysis

All the variables were entered into an electronic dataset. Descriptive analyses were performed to evaluate the distributional properties of the variables in the sample. Data are expressed as frequencies for categorial variables and mean ± standard deviation (SD) or median and interquartile range (IQR) for continuous variables, according to the normality of the distribution. A normality test was carried out to assess the normality distribution of the sample. The study population was subsequently divided into two subgroups according to the presence/absence of cPTSD. Bivariate analyses (chi-square test for categorical variables, Student’s *t*-test, or Mann–Whitney U test for continuous variables, according to the normality of the distribution) were performed to test the difference between scores at the psychometric scale in the two subgroups. A logistic regression model was used to describe the association between the presence of cPTSD as the dependent variable and PSQI subitems that scored significantly in the bivariate analyses as independent variables. We decided to use specific PSQI domains instead of the total score since the focus of the research was on sleep impairment, so we aimed to characterize these disturbances and their association with cPTSD. All tolerance values in the regression analyses were >0.1, and all variance inflation factors were <2.5, expressing that the assumption of multicollinearity was not violated. Odds ratios (ORs) and 95% confidence intervals (CI) were reported. The level of statistical significance was set at a value of *p* ≤ 0.05. Statistical analyses were performed by using the Statistical Package for Social Sciences Version 26 (SPSS, Chicago, IL, USA).

## 3. Results

Two hundred and eleven patients diagnosed with PD were consecutively recruited. Table 1 displays sample characteristics. The sample included mostly females (*n* = 108, 51.2%), with a median age of 48 years old (IQR 23). More than half of the patients were employed (*n* = 133, 63%). Moreover, most of the sample reported a positive history of comorbid lifetime psychiatric disorders (*n* = 136, 64.5%). The median age at onset was 24 years old (IQR 12), while the median age at first contact was 28 (IQR 14).

When stratifying the sample based on the ITQ score, 76 patients (36%) were diagnosed with cPTSD. Scores at the different symptom clusters characterizing PTSD, as evaluated by the ITQ, and scores concerning DSO are reported in Table 2.

Sleep quality was also assessed using the PSQI scale, from which the following results emerged: subjective sleep quality (1.75 ± 1.027), sleep latency (1.52 ± 1.114), sleep duration (1.51 ± 0.943), habitual sleep efficiency (1.43± 0.990), use of sleeping medication (1.62 ± 1.103), sleep disturbances (1.43 ± 1.041), and daytime dysfunction (1.64 ± 1.071). The mean HAM-A score was 25.60 ± 13.743 (Table 3).

No significant differences were reported in bivariate analyses between subjects with and without cPTSD for concerns of sex, age, marital status, education, and employment. Similarly, the two subgroups did not differ in terms of psychiatric familiar history, psychiatric comorbidities, age at onset, and age at first contact with psychiatric services.

As for the scores at psychopathological scales, the analysis highlighted statistically significant differences between the subgroups. Particularly, subjects with cPTSD reported significantly higher HAM-A total scores (median 43 vs. 15, *p* < 0.001). As for the DSO and PSQI scores, these were all significantly higher in the cPTSD subsample (all *p*-values < 0.001, see Table 4).

At the logistic regression, the presence of cPTSD was inserted as the dependent variable, while the PSQI scores at the subscales evaluating subjective sleep quality, sleep duration, sleep efficacy, and use of hypnotics were used as independent variables. We did not enter the PSQI scores evaluating sleep latency, sleep disturbances, and daily disturbances due to a violation of the multicollinearity assumption. After running the logistic regression model (χ^2^ = 234.220, df = 4, *p* < 0.001), the model explained between 67% (Cox and Snell’s R-squared) and 91% (Nagelkerke R-squared) of the variance. The presence of cPTSD was significantly associated with the PSQI subscores for PSQI subjective sleep quality (OR = 12.882, 95% CI 2.714–61.140, *p* = 0.001) and use of hypnotics (OR = 7.362, 95% CI 1.511–35.856) (see Table 5).

## 4. Discussion

To the best of our knowledge, this is the first study to show the association between cPTSD and sleep disorders in PD, which was associated with a higher psychopathological burden in our sample. Previous studies underlined the presence of sleep disturbances in PTSD, especially due to nocturnal nightmares [49], but such data have not been investigated in cPTSD. Indeed, sleep impairment had not yet been assessed in subjects suffering from PD who also experienced continued exposure to a traumatic event and prolonged and/or inevitable trauma, followed by avoidance, return to experience, threat feeling, dysregulation, negative self-concept, and interpersonal disturbances.

In the present study, the bivariate analyses showed higher scores at all the PSQI subitems in subjects experiencing cPTSD compared to those who did not. In addition, the cPTSD subgroup presented a higher HAM-A total score. These results confirm and corroborate what has already been reported in the literature concerning PTSD [50,51], so they were at least partially expected since sleep impairment in cPTSD was even more prevalent.

The coexistence of cPTSD and PD was associated with a worsening in sleep duration and sleep continuity. This issue could be due to the decrease in slow-wave sleep, the increase of lighter sleep stages, and disruptions in REMS that often occur in subjects who experience anxiety symptoms [52,53]. Considering the above, the alteration in different stages of sleep can lead to several neurocognitive and psychological sequelae relevant to anxiety symptomatology, such as disturbances in cognitive functions, emotion regulation, and emotional memory processing [54,55]. These findings could be exacerbated by the DSO symptoms, modifying sleep patterns, and leading to symptom worsening.

Particularly interesting is the possible link between REM sleep and dissociative symptoms. Llewellyn et al. pointed out that REM sleep is usually accompanied by associative and visual hyperactivity to encode episodic memories. The authors summarize evidence showing that during REM sleep, some brain areas (i.e., pre-frontal) are in a deactivation state, resulting in fluid reasoning and bizarre thoughts. Therefore, an excess of REM sleep or REM-sleep activity throughout the day may give cognitive functioning dissociative qualities [56]. Additionally, chronic exposure to the traumatic event seems to lead to nocturnal panic attacks emerging during the transition from lighter to deeper NREM sleep [57]. Additionally, a PD-specific sleep disturbance that can straddle panicked awakenings seen in cPTSD is night panic attacks. Nocturnal panic attacks are characterized by sudden awakenings from sleep with symptoms of panic attack [58]. These must be differentiated by sleep terrors, which are parasomnias observed in post-traumatic spectrum disturbances, leading to serious autonomic arousal and fearful behavior during NREM sleep that are not recalled in the future [59,60]. We hypothesize that the presence of cPTSD can worsen nocturnal panic attacks, possibly leading to their chronicity. An important issue raised by our results is the use of hypnotic drugs among subjects with cPTSD. This result is particularly interesting since it emphasizes the worsening of sleep symptomatology, which may determine useless attempts to control it. Although there is no information on which hypnotic medications were used by subjects in the sample, it should be underlined that previous studies do not recommend the use of BDZ in sleep disturbances due to trauma exposure [61]. Moreover, this topic is still debated, and the literature data are contradictory. In addition, the use of BDZ for a long period in patients with PTSD decreases anxiety-like behavioral and fear conditioning [62]; nevertheless, other studies demonstrated that the administration of low doses of BDZ at once after a traumatic event was associated with a worse adaptation in other stressful contexts, raising the freeze response when exposed to trauma cues [63]. Only two studies have shown the effectiveness of therapy with alprazolam and clonazepam in treating sleep disturbances in PTSD [64,65], but a large amount of literature suggests otherwise, especially during prolonged therapy. Several clinical trials demonstrated that adjunctive BDZ therapy does not ameliorate sleep quality in the short or the long term [66,67]. Several studies have shown that BDZ can interfere with fear extinction in PTSD end-delay recovery [49]. BDZ is also associated with different side effects, especially the risk of dependence and withdrawal syndrome [50]. Moreover, non-BDZ hypnotics have a better pharmacological profile and do not affect sleep architecture, but there is scant evidence in the literature that supports their use in PTSD-related nightmares and insomnia [51]. Zolpidem, compared to hypnotherapy, as an add-on therapy to an SSRI was found not to reduce sleep disturbances or other symptoms related to PTSD [68]. Indeed, prescribing BDZ in patients with PTSD and an increased risk of suicide demands particular attention [69,70]. Few data are available on pharmacological interventions in cPTSD, while psychological treatments deserve special attention in existing guidelines [71]. All things considered, the higher complexity of cPTSD treatment suggests that the same preventive measures in the use of BDZ should be implemented in this population.

Based on our results, a screening for cPTSD should be carried out for all patients who present PD and traumatic stress events in their life, and targeted drug therapy should be undertaken. Moreover, supporting an antidepressant therapy with a targeted intervention on trauma should represent the preferred therapy. In this regard, among non-pharmacological approaches for sleep disturbances, acceptance, and commitment therapy (ACT) is a third-generation cognitive behavioral therapy targeted at reducing sleep disturbance and reducing the use of BDZ [72,73]. This approach tries to emphasize psychological functions and contents by using concepts such as acceptance, mindfulness, and cognitive diffusion to modify the way people act relate to events [74,75]. Several studies described the effectivity of ACT on several mental disorders, including PTSD [76,77,78]. Recently, ACT was found to be effective in reducing the severity of insomnia, suggesting ACT can improve sleep quality and sleep disorders [75]. In this regard, ACT should be implemented in patients with sleep disturbances and a history of traumatic events.

The present study has limitations. First, the observational design in a naturalistic setting did not allow us to draw causality from the analyses. Moreover, we did not collect data on the specific medications taken by study participants. Third, due to the main aim of the study, we did not analyze and characterize the specific post-traumatic domains (e.g., emotional dysregulation) in the population affected by cPTSD. The choice of the instrument for evaluating cPTSD was based on previous literature [45], but it should be noted that the introduction of cPTSD in the current diagnostic system is relatively recent. Subsequently, the validation of further instruments that may also consider the frequency/escapability of experienced trauma could be needed in the future.

## 5. Conclusions

The results of the current study suggest that patients with PD may exhibit more severe sleep disturbances when experiencing prolonged trauma and a higher anxiety burden. Clinicians should be aware to well assess not only traumatic stress symptoms but also sleep disorders in patients suffering from PD. Further research should determine whether sleep disturbances represent a distinct phenotype exacerbated by specific DSO symptoms in these patients. Therapeutic advances, including the implementation of non-pharmacological strategies, are needed in this field.

## Figures and Tables

**Table 1:** Sample characteristics.

Sample N = 211		Median	IQR
Age (years)		38	23
Age at onset (years)		24	12
Age at first contact (years)		28	14
		**Fr**	**%**
Sex	F	108	51.2
	M	103	48.8
Comorbid psychiatric disorders	Yes	136	64.5
	No	75	35.5
Occupation	Employed	133	63.0
	Unemployed	47	22.3
	Retired	31	14.7

Abbreviations: IQR, *interquartile range*.

**Table 2:** Scores at the scales evaluating cPTSD symptoms.

ITQ Scores	Mean	SD	Median	IQR
Re-experiencing	1.34	1.501	1.00	3
Avoidance	1.47	1.516	1.00	3
Hyperarousal	1.54	1.484	1.00	3
**DSO scores**				
Affective dysregulation	1.51	1.694	1.00	4
Negative self-concept	1.16	1.438	0.00	3
Disturbances in relationships	1.40	1.622	1.00	3

Abbreviations: DSO, *disturbances in self-organization*; ITQ, *International Trauma Questionnaire*; IQR, *interquartile range*; SD, *standard deviation*.

**Table 3:** Scores at the scales evaluating sleep and anxiety symptoms.

PSQI Scores	Mean	SD
Subjective sleep quality	1.75	1.027
Sleep latency	1.52	1.114
Sleep duration	1.51	0.943
Habitual sleep efficiency	1.43	0.990
Use of sleeping medication	1.62	1.103
Sleep disturbances	1.43	1.041
Daytime dysfunction	1.64	1.071
**HAM-A scores**		
Total score	25.60	13.743

Abbreviations: HAM-A, *Hamilton Anxiety Scale*; PSQI, *Pittsburgh Sleep Quality Index*; SD, *standard deviation*.

**Table 4:** Comparison between subjects with (cPTSD) and without (no-cPTSD) complex post-traumatic stress disorder.

Socio-Demographic and Clinical Characteristics	cPTSD (n, %)	no-cPTSD (n, %)	χ^2^	*p*
Female	42 (55.3)	66 (48.9)	0.556	0.456
Paid work	50 (65.8)	88 (65.2)	0.000	1.000
Scholarity ≥ 13 years	56 (73.7)	105 (77.8)	0.253	0.615
Familiar psychiatric history	50 (65.8)	86 (63.7)	0.024	0.868
Psychiatric comorbidity	51 (67.1)	79 (58.5)	1.175	0.278
	**cPTSD** (mean, SD)	**no-cPTSD** (mean, SD)	**Student’s *t***	***p***
Age	47.07 (12.6)	46.81 (14.4)	−0.127	0.899
	**cPTSD** (median, IQR)	**no-cPTSD** (median, IQR)	**Mann–Whitney U**	***p***
Age at onset	25 (13)	24 (11)	5009.50	0.776
Age at first psychiatric contact	28.5 (14)	28 (14)	4729.50	0.345
**Psychopathological** **domains**	**cPTSD** (median, IQR)	**no-cPTSD** (median, IQR)	**Mann–Whitney U**	***p***
**DSO Symptoms**				
Affective dysregulation	4.00 (1)	0.00 (1)	0.000	*<0.001*
Negative self-concept	3.00 (0)	0.00 (0)	0.000	*<0.001*
Disturbances in relationships	4.00 (1)	0.00 (0)	2.500	*<0.001*
Total Score	10.00 (2)	1.00 (1)	0.000	*<0.001*
**PSQI Symptoms**				
Subjective quality of sleep	3.00 (0)	1.00 (1)	356.500	*<0.001*
Sleep latency	3.00 (0)	1.00 (1)	316.000	*<0.001*
Sleep duration	2.00 (1)	1.00 (0)	1376.000	*<0.001*
Sleep efficacy	2.00 (1)	1.00 (2)	1179.000	*<0.001*
Sleep disorders	2.50 (1)	1.00 (1)	574.500	*<0.001*
Hypnotics use	3.00 (0)	1.00 (2)	380.000	*<0.001*
Disorders during daytime	3.00 (0)	1.00 (1)	297.500	<0.001
Total Score	18.00 (1)	7.00 (4)	101.500	*<0.001*
**HAM-A**				
Total Score	15.00 (12)	43.00 (10)	0.000	*<0.001*

Abbreviations: DSO, *disturbances in self-organization*; HAM-A, *Hamilton Anxiety Scale*; IQR, *interquartile range*; cPTSD, *complex post-traumatic stress disorder*; PSQI, *Pittsburgh Sleep Quality Index*; SD, *standard deviation*. Significant *p*-values (<0.05) in italics.

**Table 5:** Significant associations between sleep-related variables and complex post-traumatic stress disorder.

Variables in Equation	Wald	*p*-Value	OR (95% CI)
PSQI—Sleep duration	3.050	0.081	2.741 (0.884–8.497)
PSQI—Sleep efficacy	1.405	0.236	2.426 (0.560–10.505)
PSQI—Subjective sleep quality	10.34	*0.001*	12.882 (2.714–61.140)
PSQI—Use of hypnotics	6.108	*0.013*	7.362 (1.511–35.856)

Chi-square: 234.220; df: 4; *p* < 0.001. Abbreviations: CI, *confidence interval*; OR, *odds ratio*; PSQI, *Pittsburgh Sleep Quality Index*.

## Data Availability

The data that support the findings of this study are available from the corresponding author, upon reasonable request.
